# Tumor to liver maximum standardized uptake value ratio of FDG-PET/CT parameters predicts tumor treatment response and survival of stage III non-small cell lung cancer

**DOI:** 10.1186/s12880-023-01067-6

**Published:** 2023-08-15

**Authors:** Pengfei Zhang, Wei Chen, Kewei Zhao, Xiaowen Qiu, Tao Li, Xingzhuang Zhu, Peng Sun, Chunsheng Wang, Yipeng Song

**Affiliations:** 1 Department of Radiation Oncology, Qingdao University Medical College Affiliated Yantai Yuhuangding Hospital, Yantai, 264000 Shandong China; 2 Department of Training Education, Qingdao University Medical College Affiliated Yantai Yuhuangding Hospital, Yantai, 264000 Shandong China; 3 Cancer Center, Union Hospital, Tongji Medical College, Huazhong University of Science and Technology, Wuhan, China; 4 Department of Otolaryngology, The First Affiliated Hospital of Soochow University, Suzhou, 215006 China; 5 Department of Radiation and Medical Oncology, Zhongnan Hospital of Wuhan University, Wuhan, 430071 Hubei People’s Republic of China

**Keywords:** SUVmax, Tumor-to-liver ratio, Tumor-to-blood ratio, Stage III non-small cell lung cancer, 18F-FDG PET/CT, Prognosis

## Abstract

**Background:**

To assess the predictive values of primary tumor FDG uptake for patients with inoperable stage III non-small cell lung cancer (NSCLC) after concurrent chemoradiotherapy (CCRT).

**Methods:**

A total of 107 patients with diagnosis of stage III NSCLC and CCRT were enrolled. The tumor maximum uptake value (SUVmax) was standardized by calculating several ratios between tumor and each background tissues. The receiver operating characteristics curve (ROC) was used to compare the predictive power of prognostic models. The tumor objective response rate (ORR) and overall survival (OS) were compared and analyzed by the Kaplan–Meier method and univariate and multivariate Cox regression models.

**Results:**

The areas under ROC curve (AUCs) ranged from 0.72 to 0.81 among these tumor SUVmax and standardized SUVmax ratios, and the tumor SUVmax and tumor SUVmax-to-liver SUVmean ratio (TLMR) were more predictive of ORR (AUC, 0.81; 95% CI, 0.73–0.88 for tumor SUVmax and AUC, 0.84; 95%CI, 0.76–0.91 for TLMR) than any of other SUVmax ratios. The patients with lower tumor SUVmax, SUVmean and SUVmax ratios had a significantly better OS than those with their corresponding higher ones. Moreover, both univariate and multivariable analyses revealed that TLMR was significantly associated with better ORR and OS after adjustment with other prognostic variables.

**Conclusions:**

TLMR, a standardized tumor SUVmax, was an independent prognostic predictor for tumor ORR and OS of patients with stage III NSCLC after CCRT.

## Background

The clinical stage III lung cancer accounts for approximately 8–20% of non-small cell lung cancer (NSCLC), with a median of 5-year relative survival rate of around 15.8% [1–3]. The National Comprehensive Cancer Network (NCCN) treatment guidelines suggested that concurrent chemoradiotherapy treatment (CCRT) of locally advanced lung cancer patients would have better therapeutic response and survival [4–7]. Thus, the early accurate prediction of tumor response and survival outcome may have clinical significance for optimizing the treatment strategy of this disease and result in valuable benefits to individualized treatment and improved survival and quality of life.

The 18-Fluorodeoxy-Glucose Positron Emission Tomography-Computed Tomography (^18^FDG PET/CT) allows clinicians to obtain direct and high-resolution visualization of tumor tissues with a higher level of glucose utilization and FDG uptake than the normal cells. Currently, it has become a noninvasive functional technique which can evaluate glucose metabolism at the molecular level by quantification of FDG uptake in tumor tissues. This noninvasive technique plays an increasingly important role in the diagnosis, early detection, evaluation of treatment response and early prediction of prognosis [8–11].

Several studies have indicated that the SUVmax derived from FDG- PET/CT may serve as an index of SUV measurement and prognostic predictor, which has widely accepted and most frequently used for evaluation of tumor expression activities in different types of human cancers. However, the SUVmax, as a single pixel value of PET parameters, might not be able to fully reflect glucose metabolism of the whole tumor. Furthermore, additional multiple confounding factors may also affect the reliability of SUVmax, such as insufficient correlation between systemic distribution volume and body weight, acquisition time of PET imaging, partial volume effect on positron emission tomography, leakage of administered track 18 F-FDG at injection site, susceptibility to errors in scanner calibration, loss by the injected dose decay, and selection of imaging technological parameters [12, 13]. The commonly used diagnostic ^18^ F-FDG uptake tests for background tissues, for example, the eighth hepatic segment and the blood pool of the aortic arch, have demonstrated a greatly clinical significance to minimize or reduce variability in evaluation of treatment response [14, 15]. In addition, the use of the standardized SUVs based on background tissues across different PET scanners may obtain reproducible and reliable data and more accurately reflect PET characterization of the whole tumors [16–19]. To improve prognosis and prediction ability of treatment response, some recent studies have focused on standardization of PET/CT parameters, such as tumor SUVmax-to-liver SUVmax ratio (TLR) and tumor SUVmax-to-blood SUVmax ratio (TBR) [20–27]. However, the findings from these studies on prediction of treatment response and prognosis using TLR, TBR or other ratios in NSCLC are limited. Therefore, this study using standardized PET/CT parameters was conducted to provide a systematic evaluation of predictive value for ORR and OS of patients with stage III NSCLC after CCRT.

## Materials and methods

### Patients

From June 2014 to June 2017 at the Shandong Cancer Hospital, a total of 107 consecutive patients with stage III NSCLC who underwent ^18^ F-FDG PET/CT before treatment were retrospectively enrolled in this study for evaluation of PET parameter values. Before the study, each patient was fully informed and signed written consent for this research when admitted. The Ethics Committee of the Qingdao University Medical College Affiliated Yantai Yuhuangding Hospital approved this study (ID: 2022-34), and the informed consent was obtained from each patient before this study.

An Eastern Cooperative Oncology Group (ECOG) performance status between 0 and 2 or a Karnofsky Performance Status (KPS) Scale ≥ 70 was required for all patients. The definitive diagnosis of each case was confirmed by histopathological examination. All patients were also required to meet all study inclusion criteria. All patients’ baseline characteristics were obtained from the Hospital Information System (HIS), including age, sex, smoking history, drinking history, tumor location, histology, N stage, tumor stage, CEA level, neuron specific enolase (NSE) level, Cyfra21-1 level, and ORR.

The clinicopathological characteristics of 107 patients with newly diagnosed stage III NSCLC after CCRT are summarized in Table 1. Among these patients, there were 77 males (72.0%) and 30 females (28.0%). The age of these patients ranged from 36 to 84 years, and the median age was 61 years. Of them, 36 patients had squamous cell carcinoma (SCC), and 71 patients had adenocarcinoma (ADC). The proportion of patients with smoking history (60.8%) was slightly higher than that of patients without smoking history. Of 107 NSCLC patients, 46 (43.0%) had stage IIIA disease, 61 (57.0%) had stage IIIB disease, 59 (55.1%) had central tumors, and 48 (44.9%) had peripheral tumors, respectively. Furthermore, tumor responses were sustained in 107 patients; of whom 70 patients experienced an objective CR or PR, with an overall OR rate of 65.4%, while 37 (34.6%) patients had a non-OR type of primary tumor response.

**Table 1:** Clinicopathologic characteristics

Characteristics	All cases	Percentage (%)	*P* (Objective response)
Age(years)			
≤ 65	74	69.2	0.291
> 65	33	30.8	
Sex			
Male	77	72.0	0.131
Female	30	28.0	
Smoking history			
Yes	65	60.7	0.295
No	42	39.3	
Drinking history			
Yes	59	55.1	0.514
No	48	44.9	
Tumor location			
Central	59	55.1	0.008
Peripheral	48	44.9	
Histology			
SCC	36	33.6	0.053
AC	71	66.4	
T stage			
1	22	20.5	0.522
2	34	31.8	
3	23	21.5	
4	28	26.2	
N stage			
0	6	5.6	0.361
1–3	101	94.4	
Tumor stage			
IIIA	46	43.0	0.969
IIIB	61	57.0	
CEA (median, range)	6.8	0.55–276.1	
≤ 7.60	49	45.8	< 0.001
> 7.60	58	54.2	
NSE (median, range)	13.8	5.6–66.6	
≤ 15.3	59	55.1	0.029
> 15.3	48	44.9	
Cyfra21-1(median, range)	4.94	1.04–35.6	
≤ 3.95	53	49.5	0.001
> 3.95	54	50.5	

### Treatment protocols

All patients with stage III NSCLC were treated with the combination of radiation and chemotherapy. Radiotherapy was administered daily using intensity modulated radiation therapy (IMRT) with a prescribed tumoricidal radiation dose of 60–70 Gy in 30–35 daily fractions with standard fractionation (i.e., 2.0 Gy/day except weekends). The chemotherapy was administered every 21 days in the first 2 treatment cycles simultaneously with the initial radiotherapy on Day 1 and then 4 cycles without radiation thereafter. The most frequent chemotherapy for the patients included paclitaxel, docetaxel or gemcitabine plus cisplatin.

### Acquisition and interpretation of ^18^ F-FDG PET/CT images

All study patients received the whole-body ^18^ F-FDG PET/CT by an advanced PET/CT scanner one week before treatment. Before the PET/CT examination, each patient was requested to fast and rest for approximately 6 h before injection of ^18^ F-FDG tracer (5.50 MBq/kg). No patient had a serum glucose level > 11.1 mmol/L. The PET images of patients were acquired one hour after ^18^ F-FDG injection. before PET acquisition, the non-contrast low-dose CT portion of CT scan was performed from the base of skull to the mid-thigh of the patient in the same treatment supine position on a radiolucent operation table for attenuation correction. After injection of ^18^ F-FDG tracer for 60 min, the patients underwent whole-body PET/CT scans with a field of view of 14.5 cm for 5 min and each slice thickness of axial sampling for 4.25 mm. Then the PET images were reconstructed with a three-dimensional ordered-subset expectation maximization reconstruction algorithm in a 128*128 matrix.

To define the contouring margin of the primary tumor more accurately, a region of interest (ROI) was placed over the most intense area of FDG accumulation on PET/CT images in the transaxial, sagittal, and coronal planes using a semiautomatic software for each patient. To avoid contributions from overlapping presentations with adjacent FDG-avid structures, the regions of interest were drawn carefully *via* visual inspection and manual correction of the primary tumor and a volume of interest around the tumor. The acquired standard uptake values (SUVs) were calculated based on the predefined regions of interest using the contour threshold method.

The highest SUV of the pixel in the regions of interest was recorded as the maximal standardized uptake value (SUVmax). The mean standardized uptake value (SUVmean) was obtained from the ROIs and automatically calculated by the software. At least two independent PET/CT imaging-specialized experts with more than 10-year of imaging diagnosis experience, who were blinded to the patient’s pathological and clinical information, evaluated and analyzed PET images semiquantitatively on a dedicated workstation with consensus of opinion. Two circular ROIs of 10-mm in radius were placed on the segment of VIII hepar and aortic arch (without involvement of the vessel wall) to calculate the SUVmax of the liver and the blood pool. Then the ratio of primary tumor SUVmax /liver SUVmax (TLR), primary tumor SUVmax/blood pool SUVmax (TBR), primary tumor SUVmax /liver SUVmean (TLMR), and primary tumor SUVmax/blood pool SUVmean (TBMR) were calculated.

### Evaluation of response and follow-up

All patients had a visit of follow-up with an imaging examination at outpatient clinic within the recommended schedule after completion of all CCRT therapies. The examination of the second PET-scan was not available due to economic burden and some other reasons. The assessment of objective response rate (ORR) with enhanced chest CT scans was performed within 2–4 weeks at the end of CCRT using the Response Evaluation Criteria in Solid Tumors (RECIST) Version 1.1 for therapeutic response evaluation. The overall treatment response to advanced NSCLC with CCRT was categorized as complete response (CR), partial response (PR), stable disease (SD), and progressive disease (PD). An objective response (OR) was defined as the sum of the number of patients who achieved CR or PR as their best response, while the others were defined as the non-ORs. The overall survival (OS) was defined as the time elapsed from the date of diagnosis to the date of clinical death from any cause or the last follow-up. Individuals who were alive at the end of the study or lost to follow-up were regarded as censored. During the following-up, clinical information on disease recurrence and metastasis, such as physical examination, CT, magnetic resonance imaging (MRI), and FDG PET/CT was requested every three months during the first two years and every six months for the following next three years after treatment.

### Statistical analysis

In this study, all statistical analyses were performed using the program SPSS Statistics 22.0 (SPSS Inc., Chicago, IL, USA). The Youden’s index was used to define the optimal cutoff value by maximizing the sum of the sensitivity and specificity. To estimate the optimal total number of parameter cutoffs of continuous variables for prediction of all causes of death, we conducted a preliminary analysis of predictive value of PET parameters in locally advanced NSCLC with CCRT by calculation of a receiver operating characteristic (ROC) curve. In this study, the cutoff values of SUVmax, SUVmean, TLR, TBR, TLMR, and TBMR were determined by the ROC curves regarding each survival outcome using the MedCalc Statistical Software, version 19.2 (MedCalc Software Ltd., Ostend, Belgium; https://www.medcalc.org; 2020). We drew the time-dependent ROC curves and obtained the optimal cutoff values. The range of SUVmax, SUVmean, TLR, TBR, TLMR, TBMR with Youden’s J statistic index - a maximum value of (sensitivity + specificity-1) - calculated from the ROC curves. The performance of PET/CT imaging parameters for the prediction of the tumor response was calculated by the Delong’s test to compare the areas under ROC curve (AUCs) of each index from the ROC curves.

The primary endpoint of this study was the overall survival (OS). The OS will be defined as the time from first date of treatment to the date of death from any cause or date of last follow-up. Participants who are alive at the end of the study period or lost to follow-up will be considered censored.

The Kaplan–Meier method with log-rank test was used to compare OS between each variable of SUVmax ratios. Both univariate and multivariable logistic regression Cox models were performed to estimate the hazard ratios (HRs) and corresponding 95% confidence intervals (CIs) of potential predictors of clinical tumor response to CCRT and risk of overall death.

## Results

### ROC curve analysis

**Fig. 1:**
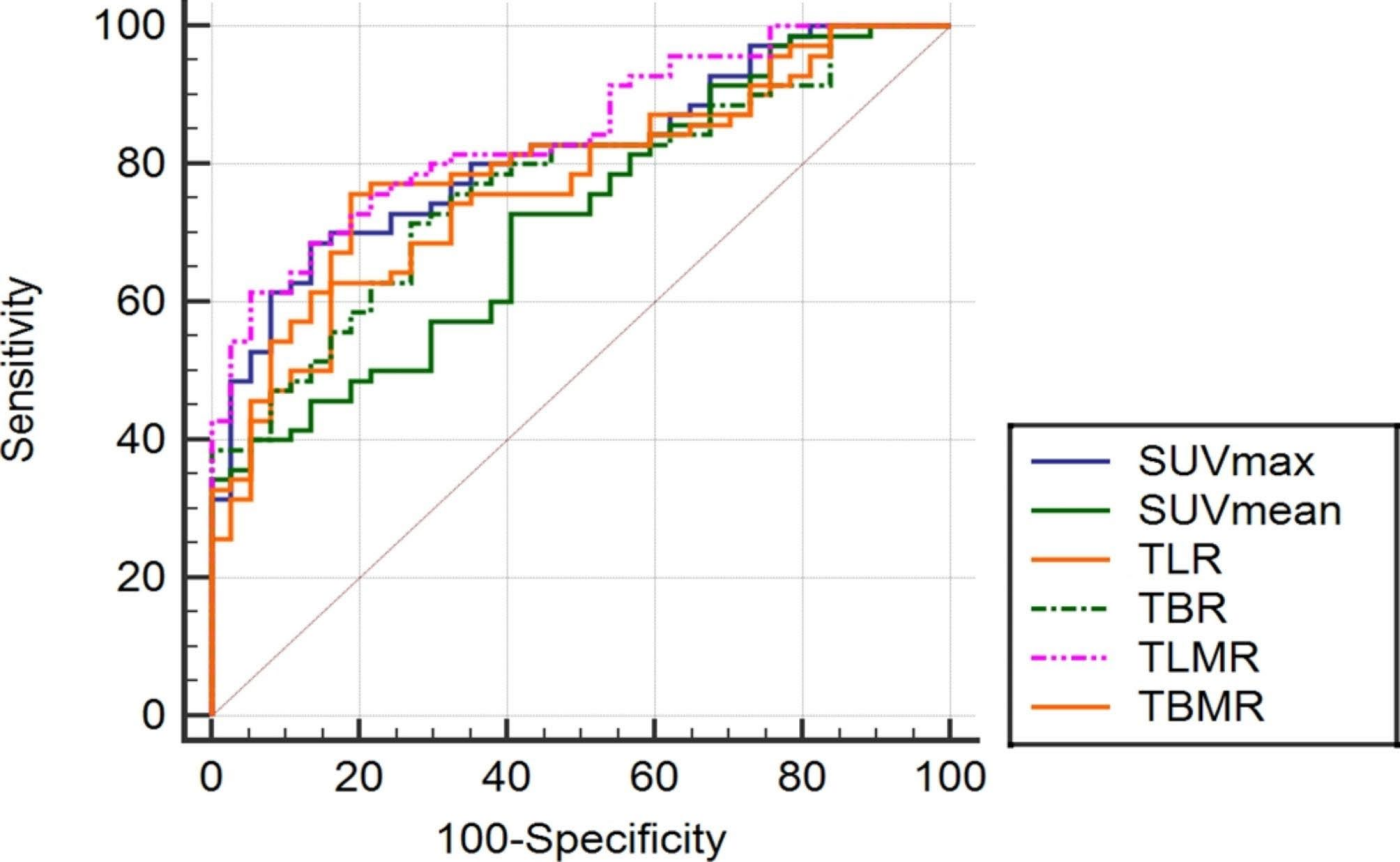
The AUCs of maximum standardized uptake of PET-derived ratios. (AUC values for TLMR, 0.84; SUVmax, 0.81; SUVmean, 0.72; TLR, 0.79; TBR, 0.77; and TBMR, 0.77, respectively)

The optimal cutoff values for SUVmax, TLR, and TLMR were 12.0, 4.27 and 4.48 with a sensitivity vs. specificity of 86.5% vs. 68.6%; 81.8% vs. 75.7%, and 94.6% vs. 61.4%, respectively as shown in Tables 2 and 3. Furthermore, the AUC of TLMR was 0.84 (95%CI, 0.76–0.91), which was significantly larger than that of SUVmax (0.81), SUVmean (0.72), TLR (0.79), TBR (0.77) and TBMR (0.77), respectively (Fig. 1).

**Table 2:** Imaging Characteristics

Characteristics	All cases	Percentage (%)	*P* (Objective response)
SUV max(3.33–28.52)			
≤ 12.0	53	49.5	< 0.001
> 12.0	54	50.5	
SUVmean(2.63–10.07)			
≤ 4.346	30	28.0	0.001
> 4.346	77	72.0	
TLR(1.13–13.71)			
≤ 4.27	60	56.1	< 0.001
> 4.27	47	43.9	
TBR(0.90-17.59)			
≤ 6.73	60	56.1	< 0.001
> 6.73	47	43.9	
TLMR(0.85–18.92)			
≤ 4.48	45	42.1	< 0.001
> 4.48	62	57.9	
TBMR(1.09–19.40)			
≤ 6.69	48	44.9	< 0.001
> 6.69	59	55.1	

**Table 3:** The ROC curves of PET parameters for predicting treatment response

Variables	AUC	SE	95% CI	△AUC	ρ	Cut-off	Sensitivity (%)	Specificity (%)
Tumor SUVmax	0.81	0.04	0.73–0.88	0.03^a^	0.16	12.1	86.5	68.6
Tumor SUVmean	0.72	0.05	0.63–0.80	0.12^b^	0.00	4.35	94.6	40.0
TLR	0.79	0.04	0.70–0.86	0.05^c^	0.00	4.27	81.1	75.7
TBR	0.77	0.05	0.68–0.85	0.07^d^	0.00	6.73	73.0	71.4
TLMR	0.84	0.04	0.76–0.91	0.07^e^	0.00	4.48	94.6	61.4
TBMR	0.77	0.05	0.68–0.85	0.04^f^	0.12	6.69	86.5	61.4

Note: ^a^SUVmax–TLMR; ^b^SUVmean–TLMR; ^c^TLR–TLMR; ^d^TBR–TLMR; ^e^TBMR–TLMR; and ^f^SUVmax–TBMR.

### Association of TLMR with treatment response

We performed both univariate and multivariable Cox regression analysis to evaluate the association of TLMR with treatment response. We found that TLMR and SUVmax were significantly associated with treatment response (cHR, 0.04; 95%CI, 0.01–0.16 for TLMR and cHR, 0.07; 95%CI, 0.03–0.21 for SUVmax) (Table 4). After adjustment with other prognostic confounders including age, sex, smoking status, drinking status, tumor location, histology, N stage, tumor stage, CEA level, NSE level, and Cyfra21-1 level, the associations remained significant (aHR, 0.09; 95%CI, 0.01–0.61 for TLMR and cHR, 0.12; 95%CI, 0.03–0.73 for SUVmax) (Table 4).

**Table 4:** Univariate and multivariable analysis on treatment response

Variables	Categories	Univariate		*P*	Multivariable		*P*
		cHR	95% CI		aHR	95% CI	
SUVmax	≤ 12.03 VS. >12.03	0.07	0.03–0.21	< 0.001	0.12	0.03–0.73	0.020
TLMR	≤ 4.48 VS. >4.48	0.04	0.01–0.16	< 0.001	0.09	0.01–0.61	0.014

Note: cHR: crude hazard ratio

aHR: adjusted hazard ratios for age, sex, smoking status, drinking status, tumor location, histology, N stage, tumor stage, CEA level, NSE level, and Cyfra21-1 level

### Association of TLMR with OS after CCRT

**Fig. 2:**
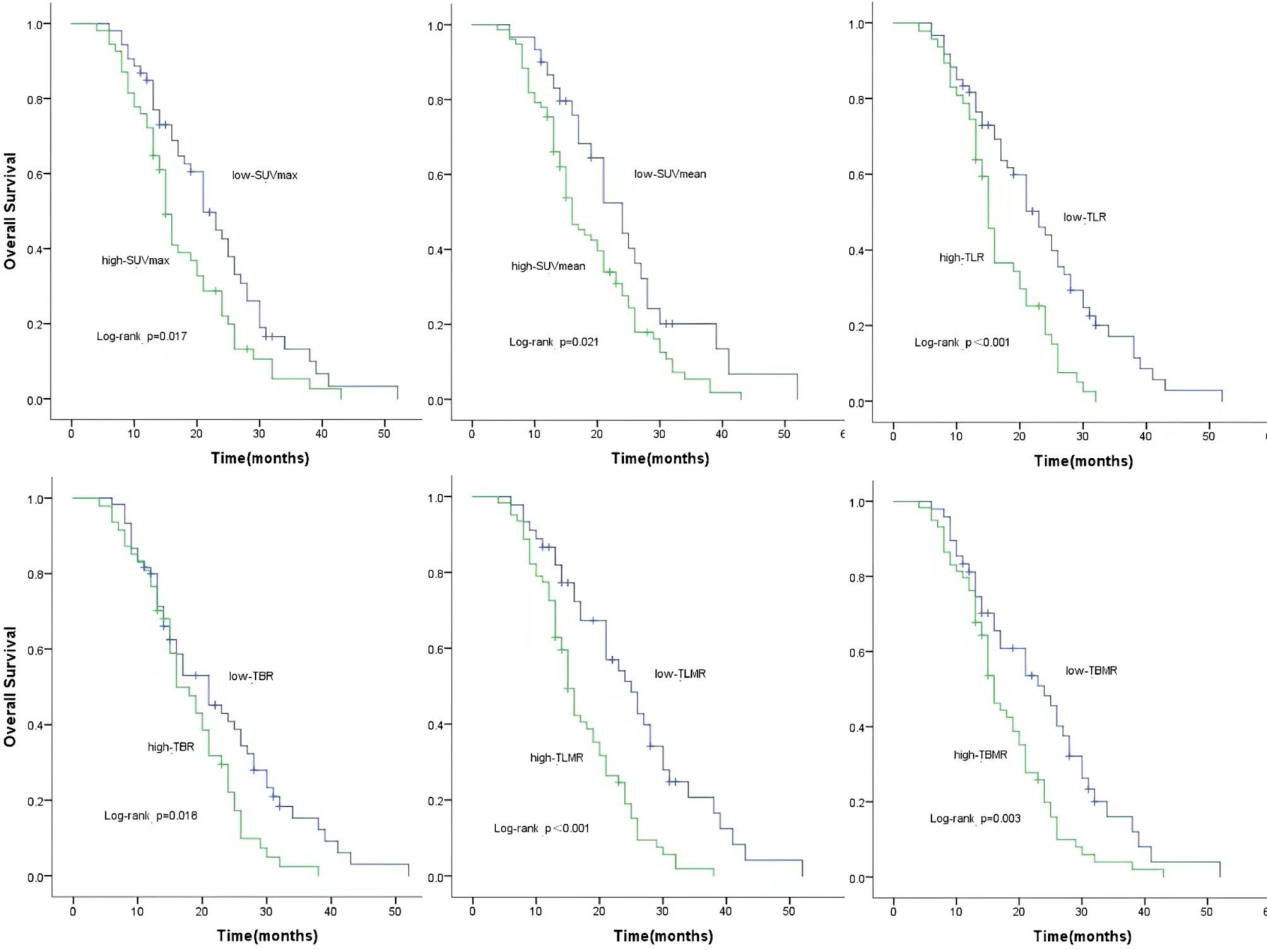
Kaplan–Meier analyses on OS according to SUVmax, SUVmean, TLR, TBR, TLMR and TBMR. (Patients with low values of SUVmax **(A)**, SUVmean **(B)**, low TLR **(C)**, low TBR **(D)**, low TLMR **(E)** and low TBMR **(F)** had better OS than those with their corresponding high values, respectively)

In this study, with the follow-up, there were 93 patients (86.9%) to die, and 14 patients (13.1%) were still alive at the end of study with a median follow up of 21.5 months. The patients with lower values of TLMR, SUVmax, SUVmean, TLR, TBR, and TBMR had significant better OS than that of corresponding higher values of each parameters (All log-rank: *P*<0.05, Fig. 2). Moreover, the patients with lower values of TLMR and SUVmax had significantly reduced risk of overall death (cHR, 0.42; 95% CI, 0.27–0.66 for TLMR and cHR, 0.62; 95% CI, 0.41–0.93 for SUVmax). Finally, we performed both univariate and multivariate Cox regression analyses to assess the associations between TLMR and SUVmax and OS of patients with *NSCLC after CCRT*. Our results showed that the lower values of TLMR and SUVmax were significantly associated with reduced risk of overall death of patients with *NSCLC after CCRT*. After controlling with other major prognostic factors including age, sex, smoking status, drinking status, tumor location, histology, N stage, tumor stage, CEA level, NSE level, and Cyfra21-1 level, we found that TLMR remained as a significantly independent prognostic predictor for *NSCLC after CCRT* (aHR, 0.45; 95% CI, 0.24–0.85 for TLMR and aHR, 0.85; 95% CI, 0.49–1.49 for SUVmax (Table 5).

**Table 5:** Univariate and multivariable analysis on overall survival (OS)

Variables	Categories	Univariate		*P*	Multivariable		*P*
		cHR	95%CI		aHR	95%CI	
SUVmax	≤ 12.02 VS. >12.02	0.62	0.41–0.93	0.022	0.85	0.49–1.49	0.574
TLMR	≤ 4.48 VS. >4.48	0.42	0.27–0.66	< 0.001	0.45	0.24–0.85	0.014

Note: cHR: crude hazard ratio

aHR: adjusted hazard ratios for age, sex, smoking status, drinking status, tumor location, histology, N stage, tumor stage, CEA level, NSE level, and Cyfra21-1 level

## Discussion

CCRT is the main treatment for patients with stage III NSCLC according to the NCCN treatment guidelines [7], while the treatment response and OS of these patients after CCRT differ individually. Thus, the early prediction of tumor treatment response becomes so important that the clinicians may identify those patients who may be sensitive or insensitive to CCRT before treatment and can guide the formulation for more accurate and personalized treatment strategies. In the current study, we conducted this retrospective study to explore the potential predictive value of TLMR in PET-based baseline parameters for the tumor response and OS of patients with NSCLC after CCRT, and we demonstrated that pretreatment parameter TLMR was an independent predictive factors of clinical tumor response and OS. This study showed that the patients had lower TLMR values prior to CCRT achieved a better treatment response and OS than those with higher value. This study also demonstrated that TLMR was an independent prognostic predictor of OS for these patients. To the best of our knowledge, this is the first study to examine the prognostic value of TLMR ratio, which is calculated from PET/CT parameters between tumor lesions and the reference background in patients with locally advanced NSCLC after CCRT.

^18^FDG-PET/CT, which is a tool to evaluate tumor metabolism and reveal the uptake of FDG by tumor cells, has been applied widely for early detection of tumor locations and the decision-making of anticancer intervention. Some previous studies have reported that several baseline parameters derived from PET/CT before initiation of treatment, such as SUVmax and SUVmean might predict clinical treatment response and recurrence in patients with various types of human cancers [28–31]. A previous retrospective study of stage III NSCLC patients showed that SUVmax significantly affected the OS of patients with NSCLC, which is consistent with our finding that the patients with a higher SUVmax (SUVmax ≥ 14) had a worse OS than those with a lower SUVmax (SUVmax < 14) (median of OS: 18 months vs. 25 months) after definitive chemoradiotherapy [32]. Kanyilmaz et al. found that SUVmax had prognostic value in 103 patients with stage III NSCLC receiving ^18^FDG-PET-CT between 2010 and 2017 [33]. Furthermore, a retrospective study of 73 stage III NSCLC patients receiving concurrent definitive chemoradiotherapy indicated an association between SUVmax and complete treatment response and OS. This study showed that patients with a higher SUVmax (≥ 12) had a lower CR rate of 19% and a worse OS of 21 months than those with a lower SUVmax (< 12) (CR: 19% vs. 60% and OS: 21 months vs. 26 months) [34]. Taken together, these results indicate that primary tumor SUVmax of PET/CT may predict treatment response and OS for the patients with stage III NSCLC after definitive CCRT.

However, the use of SUVmax as a predictor has some disadvantages: 1) It is only a single-pixel value and is relatively vulnerable to statistical noise. 2). It may be affected by the differences in body part composition and habitat, as well as by time-dependent factors. The results from SUVmax may indicate the highest uptake of FDG in the tumor rather than the overall burden or metabolic activity of the tumor. 3) In addition, there are some technical errors in SUVmax caused from the PET scanner calibration, image reconstruction methods used by different institutions, and imaging correction protocols. Therefore, the use of SUVmax only as a surrogate marker may not adequately represent metabolic reaction of primary tumors.

To overcome several limitations of SUVmax, the volume-based PET parameters such as metabolic tumor volume (MTV) and total lesion glycolysis (TLG) have been widely used in clinical practice. Compared with SUVmax, MTV and TLG have shown promise as prognostic factors for NSCLC and may serve as predictors of prognosis, while they have a major potential problem with the lack of consensus for researchers to define the SUV threshold.

In most of recent years, some researchers found that as the reference background tissues could maintain a nearly constant level of SUV index either between or after injections of ^18^ F-FDG over time, it could be likely to be used for better prediction of patients’ prognosis. In many types of metabolic parameters, some studies have shown that the normal liver and blood pool tissues are the most frequently used as normal candidate ones in metabolic parameters compared with other organs [15, 35, 36]. Domenico et al. using SUVmax, lesion-to-liver SUVmax ratio and lesion-to-blood pool SUVmax ratio investigated the particular metabolic behavior of lymphoma and found that only TLMR (the tumor-to-liver SUVmax ratio) was an independent factor associated with the Ki-67 score [37]. Another study found that the normalized uptake of SUVmax to liver has the most significant impact on the pathologic complete response in patients with locally advanced rectal cancer after receiving neoadjuvant chemotherapy [15]. Seunghyeon et al. found that the standardized uptake tumor-to-blood ratio was an independent predictor of recurrence (p = 0.0014) in patients with NSCLC, with an AUC of 0.76 for TBR at a cutoff value of 4.0 [38]. Moreover, several recent studies have shown that TLR achieved a superior reflection of whole-tumor metabolic activity compared to SUVmax, which not only reflects a single voxel value of the highest uptake of FDG within a tumor but also represents the total tumor burden and metabolic activity [29, 39, 40]. For example, the TLR in esophageal cancer was superior to tumor SUVmax as an independent prognostic index for OS and as a predictive value for distant metastasis. The patients with a higher TLR (HR, 21.9; 95% CI: 2.3–213.0; P = 0.008) were more likely to have a poor treatment response. Similarly, the patients with a higher level of TLR experienced significantly shorter OS than those with a lower level of TLR (median OS: 13.5 months vs. 19.30 months) [28].

There is some evidence of potential advantages of TLMR as a prognostic predictor. For example, it is an independent pretreatment index after adjustment of different confounding factors such as weight, management activities, and clinical operation of PET/CT instruments. The above factors in SUV methodology and the inherent system variability can be reduced by the standardized protocol for different PET/CT scanners. For practical clinical use, it is a relatively simple technology in daily clinical practice so that the investigators can intuitively identify primary tumor uptake from the screen compared to that in background tissue, such as liver or blood pool without any additional requirements, special software, and costly equipment. In this analysis, we have provided a unique method to enable researchers not only to adjust their analysis and ensure reproducible results, avoiding additional procedures or the need for further diagnostic imaging, but also not to cause additional economic burden and radiation exposure. In this current study, with the normalization of SUVmax to the liver or blood pool, TLMR has been demonstrated to have a significant prognostic value in our patients with NSCLC after CCRT. Furthermore, compared to SUVmax, the use of TLMR may avoid some SUV-specific systematic and metabolic errors. The following reasons may explain the discrepancy, such as only a dimensionless quantity of metabolic index, vulnerable FDG uptake TLMR is not prone to potentially serious errors in the patients with different glucose levels compared with SUVmax. As demonstrated, TLMR rather than SUVmax may play a potentially important role in assessment of primary tumor metabolism and prognosis. Several potential limitations should be considered when interpreting the results from our current study, including a retrospective study, small numbers of study patients included, imaging data rather than confirmed diagnosis by pathology, and the nature of its single hospital-based design.

In Conclusion, PET-derived TLMR may be superior to tumor SUVmax as a more valuable predictor for treatment response and OS in patients with NSCLC after CCRT. The patients with a lower TLMR are more likely to have a better treatment response and OS after CCRT than those with a higher TLMR. Such findings may help clinicians better make assessment for patients with stage III NSCLC before CCRT.

## Data Availability

The datasets used and/or analyzed during the current study are available from the corresponding author on reasonable request.

## Abbreviations

FDG: ^18^ F-fluorodeoxyglucose
PET: Positron Emission Tomography
CT: Computed Tomography
MRI: Magnetic Resonance Imaging
^18^FDG PET/CT: 18-Fluorodeoxy-Glucose Positron Emission Tomography-Computed Tomography
NSCLC: Non-small cell lung cancer
SCC: Squamous cell carcinoma
ADC: Adenocarcinoma
CCRT: Concurrent chemoradiotherapy
CEA: Carcinoembryonic antigen
NSE: Neuron specific enolase
ECOG: Eastern Cooperative Oncology Group
AJCC: American Joint Committee on Cancer
KPS: Karnofsky Performance Status
HIS: Hospital Information System
M0: No distant metastasis
IMRT: Intensity modulated radiation therapy
ROI: Region of interest
AUC: Area under the ROC curve
SUVs: Standardized uptake values
SUVmax: Maximum standardized uptake value
SUVmean: Mean standardized uptake value
MTV: Metabolic tumor volume
TLG: Total lesion glycolysis
TLR: Tumor SUVmax-to-liver SUVmax ratio
TBR: Tumor SUVmax-to-blood SUVmax ratio
TLMR: Tumor SUVmax-to-liver SUVmean ratio
TBMR: Tumor SUVmax-to-blood SUVmean ratio
RECIST: Response Evaluation Criteria in Solid Tumors
CR: Complete response
PR: Partial response
SD: Stable disease
PD: Progressive disease
OR: Objective response
ROC: Receiver operating characteristic
ORR: Objective response rate
OS: Overall survival
CI: Confidence interval
HR: Hazard ratio
